# The left atrial substrate plays a significant role in the development of complex atrial tachycardia in patients with precapillary pulmonary hypertension

**DOI:** 10.1186/s12872-019-1142-z

**Published:** 2019-06-28

**Authors:** Zdenka Fingrova, Stepan Havranek, David Ambroz, Pavel Jansa, Ales Linhart

**Affiliations:** 1 2nd Department of Medicine - Department of Cardiovascular Medicine, General University Hospital in Prague, U Nemocnice 2, 12808 Prague, Czech Republic; 2 2nd Department of Medicine - Department of Cardiovascular Medicine, 1st Faculty of Medicine, Charles University in Prague, U Nemocnice 2, 12808 Prague, Czech Republic

**Keywords:** Pulmonary hypertension, Atrial fibrillation, Atrial tachycardia, Atrial flutter

## Abstract

**Background:**

Atrial fibrillation (AF) and related atrial tachyarrhythmias (AT), including type I atrial flutter (AFL) are frequently observed in patients with pulmonary hypertension (PH). Their relationship to hemodynamic changes, atrial size, and ventricular function are still not fully verified.

**Methods:**

We retrospectively studied hemodynamic data, echocardiographic findings and arrhythmia incidence in 814 patients with invasively diagnosed precapillary PH (aged 59 ± 14 years; 46% males). Patients with combined or post-capillary PH were excluded.

**Results:**

AF / AT were identified in 225 (28%) of all the study population. Compared to the subgroup without arrhythmia, patients with AF / AT had elevated right atrial pressure (11 ± 5 vs. 9 ± 5 mmHg), wedge pressure (11 ± 3 vs. 10 ± 3), a more enlarged right atrium (50 ± 12 vs. 47 ± 11 mm) and an increased left atrial diameter in the parasternal long axis projection, *p* <  0.05 for all comparisons. In the multivariate model, the left atrial size, patient age, arterial hypertension, diabetes and type of PH were associated with AF / AT occurrence, *p* <  0.05. Patients with type I AFL were more frequently male (39 (80%) vs. 62 (42%)), were younger (61 ± 11 vs. 67 ± 10 years), had increased pulmonary artery mean pressure (50 ± 12 vs. 45 ± 12 mmHg), less advanced left atrial dilatation (38 ± 10 vs. 42 ± 7 mm), and a more enlarged right atrium (56 ± 12 vs. 48 ± 11) as compared to subjects with AF or other AT, *p* <  0.05.

**Conclusions:**

The evidence of elevated wedge pressure and the enlargement of the left atrium especially in patients with AF suggest a parallel involvement of the left atrial substrate in arrhythmia formation despite invasively confirmed evidence of purely isolated precapillary PH. Substantial differences were noticed between patients with type I AFL and the remaining patients with other arrhythmia types.

## Background

Pulmonary hypertension (PH) is a pathophysiological disorder characterized by an elevated pulmonary artery mean pressure (PAMP) ≥ 25 mmHg [1]. Hemodynamic parameters evaluated by a right heart catheterization defined the precapillary PH, isolated postcapillary or combined post- and precapillary PH [1, 2]. Despite advances in the management of patients with PH, most treatment strategies relieve symptoms and functional status while evidence of prognostic improvements and survival is limited [1].

Supraventricular tachycardias has been frequently (range of cumulative incidence 10–25%) observed in patients with idiopathic pulmonary arterial hypertension (PAH) [3], in all types of PH [4–6], inoperable chronic thromboembolic pulmonary hypertension (CTEPH) [4, 6] or Eisenmenger syndrome [7]. Atrial tachyarrhythmias leads to clinical deterioration and may be associated with an increased risk of death [3, 4, 6, 8]. Out of all types of supraventricular tachycardias in the PH population, atrial fibrillation (AF) and related atrial tachyarrhythmias (AT), including type I atrial flutter (AFL) are the most frequently observed [3–7].

It is known that there is a relationship between right atrium (RA) enlargement in patients with PH and the increased prevalence of supraventricular arrhythmia [9], however, limited data is available on the arrhythmogenic substrate for complex ATs including AF in patients with precapillary PH. Although some studies in patients with PH or respiratory disease suggested that substrate for AF / AT could be predominantly situated in the RA [10–13], the role of the arrhythmogenic substrate in the left atrium (LA) and left atrial pressure in patients with precapillary PH is generally unknown.

In order to further evaluate the role of the left and right atrial substrate in the development of AF / AT in patients with precapillary PH, we conducted a retrospective analysis of records focused on left and right cardiac morphology and haemodynamics in patients with precapillary PH and AF / AT.

## Methods

Consecutive unselected patients, who were diagnosed and treated for PH at a single centre between 2003 and 2017, were enrolled in the retrospective analysis. Patient data was retrieved from a dedicated registry. The study was performed according to good clinical practice and in compliance with the Helsinki declaration. An individual written consent was obtained from each patient. The study was approved by the local Ethics committee.

All patients have undergone a complete routine baseline in-hospital work-up according to contemporary standards [1, 10, 11] including a medical history assessment, concomitant diseases, clinical severity, functional capacity, complete laboratory tests, echocardiography, and other noninvasive and invasive methods. All included patients underwent a right heart catheterization demonstrating PH with a pulmonary artery mean pressure (PAMP) ≥ 25 mmHg. Only patients with precapillary PH with a pulmonary artery wedge pressure (PAWP) ≤ 15 mmHg were included in the current analysis. The classification of PH and patient management was done according to current European Society of Cardiology guidelines [2]. Patients with PAH, PH due to lung diseases and/or hypoxia, CTEPH and a group of PH with unclear and/or multifactorial mechanisms were enrolled in the study.

For patients with CTEPH, assessment of operability included perfusion scintigraphy and pulmonary angiography and when eligible, pulmonary endarterectomy (PEA) was indicated.

All patients were regularly seen at 1 to 6 monthly intervals, or whenever clinically indicated, in an outpatient clinic. Standard 12-lead ECG were obtained as part of a regular follow-up program.

Prevalent AF / AT (common, type I AFL included) was defined as the presence of arrhythmia on 12-lead surface ECGs, 24-h ECG monitors and / or during invasive electrophysiology testing and / or as indicated by a diagnosis found in the medical records, hospitalization or ambulatory databases. The diagnosis of AF / AT was confirmed by an experienced cardiologist.

Based on clinical experiences and referred guidelines, rhythm control, i.e. the restoration of the sinus rhythm was usually attempted in all patients with symptomatic or clinically significant tachycardia which was not previously classified as permanent, irrespective of the heart rate and underlying conditions. Patients with previously documented, known paroxysmal or persistent AF or other AT than type I AFL were treated with electrical cardioversion, if the sinus rhythm was not restored spontaneously or after initial antiarrhythmic therapy or in cases of heart failure symptoms. When symptomatic recurrent AF / AT was manifested, a catheter ablation (CA) was scheduled. In cases of type I AFL, the primary strategy was to restore the sinus rhythm with an early CA and whatever seemed appropriate in the given clinical context. Electro-anatomical mapping (CARTO 3, Biosense-Webster Inc., Diamond Bar, CA, USA) was used in several cases when a more complex arrhythmia or arrhythmogenic substrate was predicted. In that case, manual catheter navigation was used for reconstruction of the atrial endocardial surface. Uniformly distributed mapping points were acquired at sites with stable endocardial contact. Special attention was paid to make sure mapping points were not included behind the pulmonary vein ostia. The orifice and proximal part of LA / RA appendage was always mapped. Precise delineation of the mitral annulus was performed in all cases. Intracardiac echocardiography was used to visualize and tag critical structures. 3D dense bipolar voltage maps of the atria were built (> 100 points). Low-voltage regions were defined as bipolar voltage < 0.1 mV. The size of low-voltage areas was rated as a proportion of the surface area with reduced bipolar voltages from the whole atrial surface (mitral and tricuspidal annuli were excluded). Atrial volume was assessed using a built-in computation function of the Biosense system.

### Statistical analysis

Continuous variables were expressed as means with standard deviations after testing for normality (Shapiro-Wilk’s test) and compared with the 2-tailed t-test for independent samples or advanced ANOVA tests. Categorical variables were expressed as percentages and compared with the χ2–test or Kruskal-Wallis test when appropriate. Multivariate analysis was used to identify independent predictors of mortality. *P*-value < 0.05 was considered significant. All analyses were performed using the STATISTICA vers.12 software (Statsoft, Inc., Tulsa, USA).

## Results

A total of 814 patients (aged 59 ± 14 years; 46% males) were analysed. Baseline characteristics of the total population and subgroups are shown in Table 1. Arrhythmia was documented in 225 (28%) of the subjects. Patients with a history of AF / AT were older, had more frequently arterial hypertension, diabetes mellitus and were treated with a specific therapy. Out of all patients with arrhythmia, AF was manifested in 149 (66%) and AT in 76 (34%) subjects, respectively. Type I AFL was diagnosed in 49 patients (64% of all subjects with AT). An excessive prevalence of AF / AT was noticed among patients with CTEPH (AF: 49 (55%), AFL: 28 (31%), other AT: 11 (12%)), and more specifically when patients were treated by PEA (AF: 25 (64%), AFL: 20 (40%), other AT: 6 (12%)).

**Table 1:** Baseline clinical and demographical data

	Total (*n* = 814)	Arrhythmia YES (*n* = 225)	Arrhythmia NO (*n* = 589)	*p*
Age (years)	59 ± 14	65 ± 11	57 ± 15	< 0.001
Males	378 (46%)	113 (50%)	265 (45%)	NS
Group 1 PH: PAH	334 (41%)	87 (39%)	247 (41%)	NS
Group 3 PH: Lung diseases	140 (17%)	23 (10%)	117 (20%)	0.0007
Group 4 PH: CTEPH	303 (37%)	106 (47%)	197 (33%)	0.0002
Group 5 PH: Other types of PH	37 (5%)	9 (4%)	28 (5%)	NS
Arterial hypertension	470 (58%)	160 (71%)	310 (53%)	< 0.0001
Diabetes mellitus	204 (25%)	74 (33%)	130 (22%)	0.03
Coronary artery disease	168 (21%)	49 (22%)	119 (20%)	NS
Chronic obstructive pulmonary disease / obstructive sleep apnea	195 (23%) / 49 (6%)	62 (28%) / 10 (4%)	133 (22%) / 39 (7%)	NS
Specific therapy	416 (51%)	109 (48%)	307 (52%)	< 0.01
PEA in CTEPH	173 (21%)	68 (30%)	105 (18%)	0.0002
NYHA	2.69 ± 0.79	2.66 ± 0.81	2.71 ± 0.78	NS
6-min walking test (m)	341 ± 129	329 ± 127	346 ± 129	NS
Mortality	379	140 (49%)	320 (48%)	NS

Legend: Values are expressed as Mean ± SD or n (%). *PH* Pulmonary hypertension, *PAH* Pulmonary arterial hypertension, *CTEPH* Chronic thrombembolic pulmonary hypertension, *PEA* Pulmonary endarterectomy

**Fig. 1:**
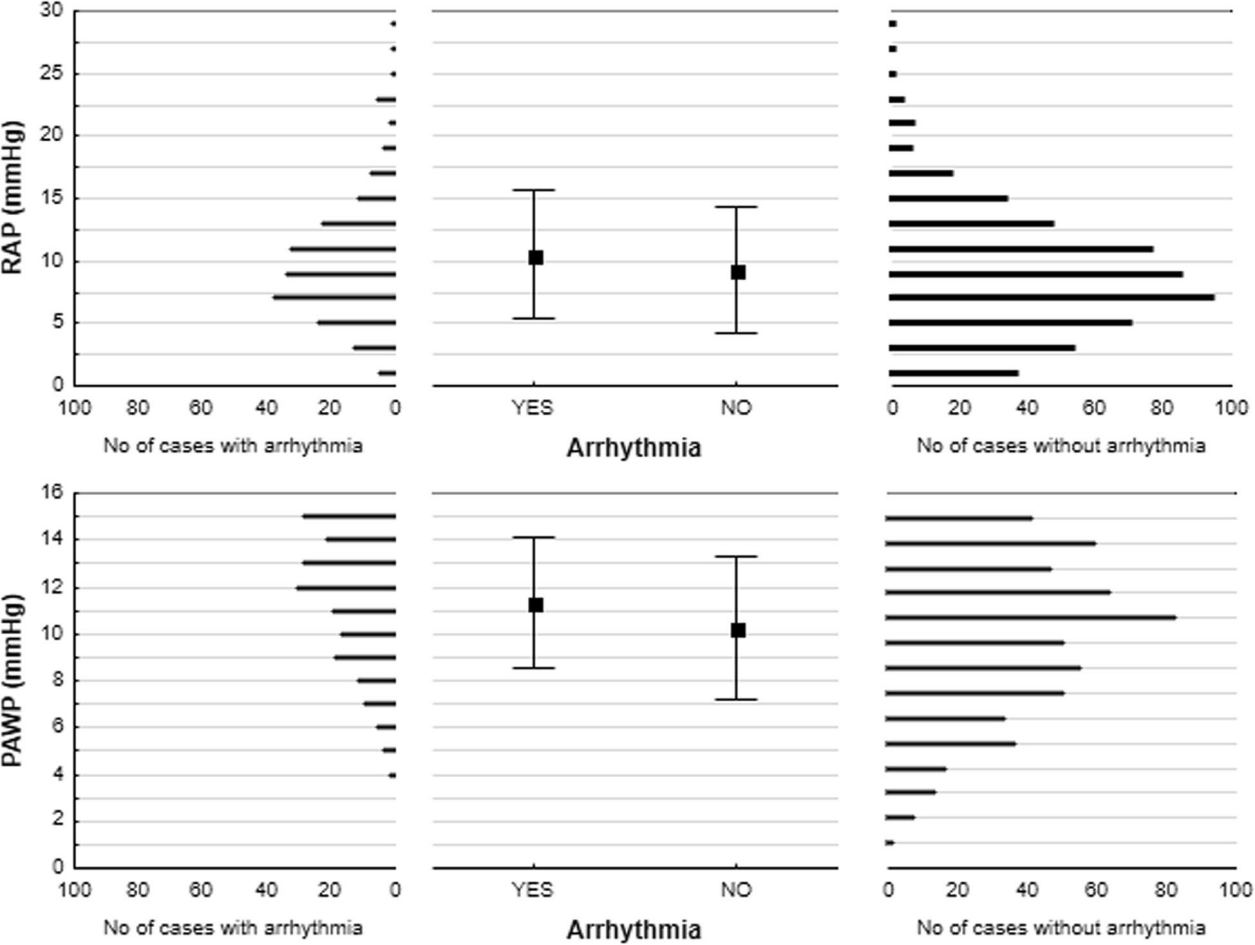
Distribution of right atrial pressure and pulmonary artery wedge pressure in relation to arrhythmia manifestation. PAWP – pulmonary artery wedge pressure; RAP – right atrial pressure

Patients with a history of AF / AT had significantly larger LA diameters, end-diastolic left ventricular (LV) diameter and RA short diameter estimated with 2D echocardiography. In the same group, the PAWP and RA pressure (RAP) were also elevated as compared to patients without arrhythmias, Table 2 and Fig. 1. In contrast, there was no significant difference in right ventricular (RV) diameters, tricuspid annular plane systolic excursion (TAPSE) and LV ejection fraction when patients with and without AF / AT were compared. For more details see Table 2. When comparing patients who already had existing arrhythmia at the time of the PH diagnosis of PH with those with new-onset of AF / AT during a follow-up, no significant difference in the LA diameter was recognized (46 ± 8 vs. 44 ± 8 mm; *p* = 0.3). Patients with paroxysmal forms and persistent / permanent forms of arrhythmia had LA diameters without any variance (46 ± 7 vs. 48 ± 8 mm; *p* = 0.1).

**Table 2:** Echocardiographical and heamodynamical data

	Arrhythmia	Arrhythmia	*p*
	YES	NO	
	(n = 225)	(n = 589)	
LA diameter / PLAX (mm)	44 ± 8	40 ± 10	< 0.001
LA long / A4C (mm)	55 ± 9	50 ± 8	< 0.0001
LA short / A4C (mm)	40 ± 8	36 ± 8	< 0.0001
RA short / A4C (mm)	50 ± 12	47 ± 11	< 0.01
RV short / A4C (mm)	46 ± 10	45 ± 10	NS
TAPSE (mm)	18 ± 5	18 ± 5	NS
LV enddiastolic diameter (mm)	47 ± 8	44 ± 8	< 0.01
LV EF (%)	62 ± 9	63 ± 8	NS
PAMP (mm Hg)	47 ± 13	49 ± 16	NS
RAP (mm Hg)	10.5 ± 5.1	9.3 ± 5.1	< 0.01
PAWP (mm Hg)	11.4 ± 2.8	10.2 ± 3.1	< 0.01

Legend: Values are expressed as mean ± SD. *LA* Left atrium, *RA* Right atrium, *RV* Right ventricle, *LV* Left ventricle, *TAPSE* Tricuspid annular plane systolic excursion, *EF* Ejection fraction, *PAMP* Pulmonary artery mean pressure, *RAP* Right atrial pressure, *PAWP* Pulmonary artery wedge pressure, *PLAX* Parasternal long axis projection, *A4C* Apical four chamber projection

In a multivariate model of the LA diameter in a parasternal long axis projection; age, arterial hypertension, diabetes and type of PH were associated with the occurrence of AF / AT (Table 3).

**Table 3:** Multivariate analysis. Prediction of arrhythmia occurrence

Parameter	F	*p*
Age (years)	5.1	0.02
Arterial hypertension	6.0	0.015
Diabetes mellitus	7.1	0.01
Occurrence of PAH	5.1	0.0001
Specific therapy	3.0	NS
LA diameter in PLAX (mm)	5.1	0.02
RA diameter in A4C (mm)	0.8	NS
LV enddiastolic diameter (mm)	2.5	NS
RA pressure (mmHg)	0.02	NS
PAWP (mmHg)	0.1	NS

Legend: Significant variables from Tables 1 and 2 were included. *PH* Pulmonary hypertension, *LA* Left atrium, *RA* Right atrium, *LV* Left ventricle, *PAWP* Pulmonary artery wedge pressure, *PLAX* Parasternal long axis projection, *A4C* Apical four chamber projection

**Fig. 2:**
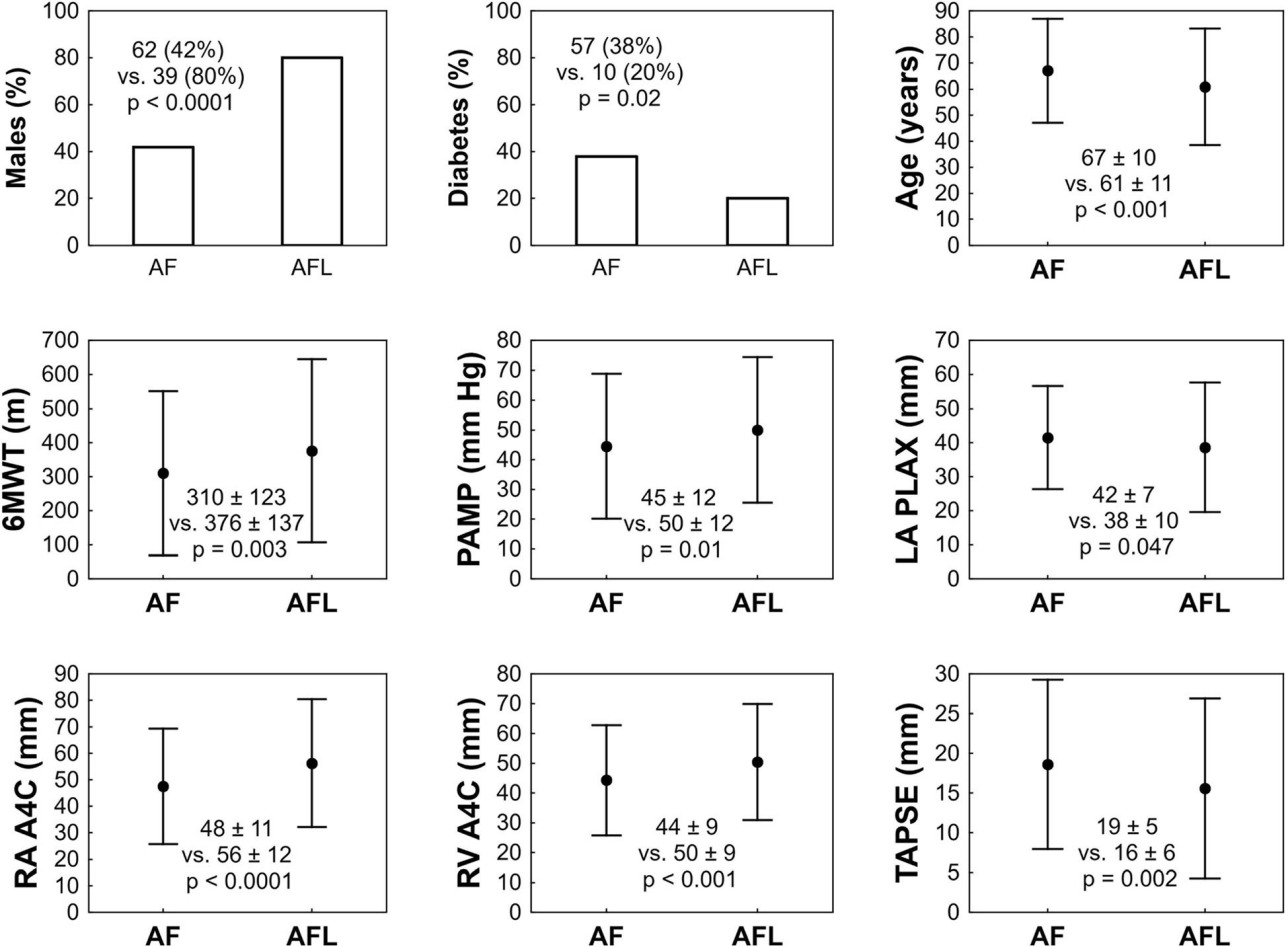
Clinical, demographical, haemodynamic and echocardiographic data in patients with atrial fibrillation and flutter. Values are expressed as mean ± standard deviation or as n (%). AF – atrial fibrillation; AFL – atrial flutter; 6MWT – six-minute walking test; PAMP – pulmonary arterial mean pressure; PAWP – pulmonary arterial wedge pressure; LA – left atrium; RA – right atrium; RV – right ventricle; TAPSE – tricuspid annular plane systolic excursion

Out of all patients with arrhythmia, 49 (22%) subjects manifested type I AFL. When compared to patients with type I AFL, subjects with AF or other AT were more frequently female, had more prevalent diabetes, were older, manifested more reduced 6-min walking test distance, lower PAMP values, advanced LA dilatation, smaller RA and RV diameters and better TAPSE. More details are shown in Fig. 2. In addition, patients with AFL manifested higher values of RAP then patients with AF (12 ± 6 vs. 9 ± 5 mmHg; *p* = 0.01). After excluding AFL patients, the differences in RAP between AF patients and patients without arrhythmia are nonsignificant (9 ± 5 vs. 10 ± 5 mmHg; *p* = 0.1). The PAWP values were comparable between AFL and AF patients (11 ± 3 vs. 12 ± 3 mmHg, *p* = 0.15).

**Fig. 3:**
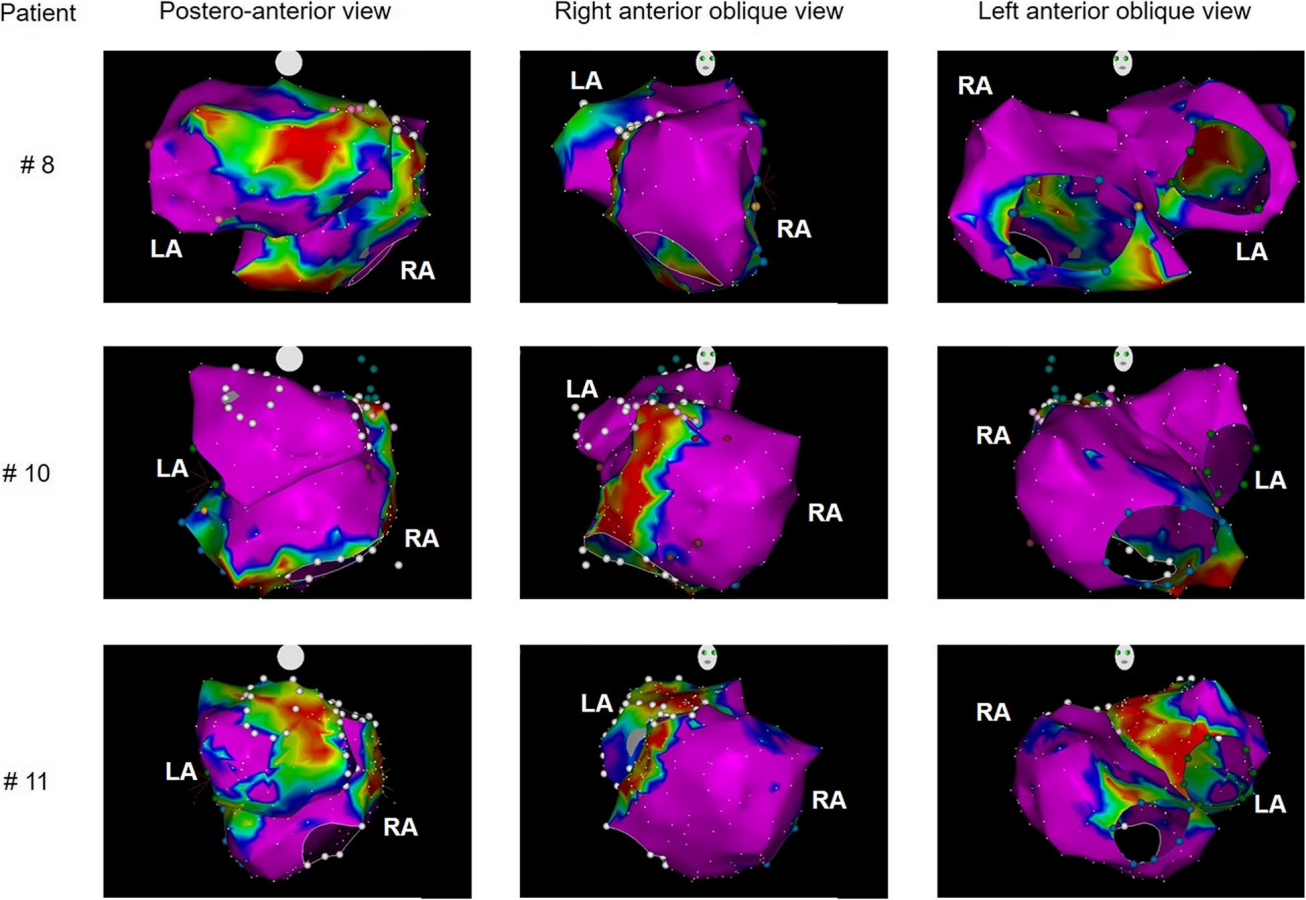
An example of RA and LA arrhythmogenic substrate. Example of 3D electro-anatomical bipolar voltage maps of both right and left atrium (see Table 4 for more individual details). Red colour represents areas with reduced bipolar voltage (< 0.1 mV). LA – left atrium; RA – right atrium

Out of 45 patients treated with CA, electro-anatomical mapping was used in 11 (24%) cases. Overall 9 patients (20%) manifested common AFL as a clinical diagnosis. One patient had both AFL and a non-specified AT. One patient manifested AF and a different AT. And 1 patient had AF. In all patients an electro-anatomical map of RA was performed. In three cases left atrial mapping was performed as well. More details are listed in Table 4 and Fig. 3.

**Table 4:** Pilot data of LA / RA morphology in some patients after catheter ablation with the use of electro-anatomical mapping

# Case	Gender	Age (years)	Type of PH	Type of arrhythmia	RAP (mm Hg)	RA short (mm)	PAWP (mm Hg)	LA v PLAX (mm) / LAVi (ml/m^2^)	RA CARTO volume (ml)	RA surface / low voltage surface (cm^2^)	LACARTO volume (ml)	LA surface / low voltage surface (cm^2^)
1	M	73	CTEPH	AFL	6	63	8	55 / 41	180	196 / 1	–	–
2	M	81	CTEPH	AFL	9	40	12	54 / 41	151	166 / 20	–	–
3	F	72	CTEPH	AFL	8	45	13	43 / 44	183	175 / 1	–	–
4	M	75	Hypoxic	AFL	8	54	3	41 / 22	242	228 / 31	–	–
5	F	81	CTEPH	AF / AT	6	38	13	38 / 30	191	198 / 9	–	–
6	F	74	Hypoxic	AFL	9	66	11	56 / 47	184	198 / 38	–	–
7	M	59	PAH	AFL	20	77	9	52 / 30	276	267 / 2	–	–
8	M	74	CTEPH	AFL	19	51	12	53 / 35	206	202 / 5	180	182 / 2
9	M	61	CTEPH	AFL	14	56	10	40 / 52	217	228 / 33	–	–
10	M	46	PAH	AFL / AT	13	77	6	40 / 12	395	296 / 26	90	123 / 0
11	M	87	CTEPH	AF	6	64	14	56 / 73	324	174 / 16	173	169 / 18

Legend: *M* Male, *F* Female, *RAP* Right atrial pressure, *RA* Right atrium, *PAWP* Pulmonary artery wedge pressure, *LA* Left atrium, *PLAX* Parasternal long axis projection, *LAVi* Indexed left atrial volume, *CTEPH* Chronic thromboembolic pulmonary hypertension, *AFL* Atrial flutter type I, *AF* Atrial fibrillation, *AT* Atrial tachycardia, *PAH* Pulmonary arterial hypertension

## Discussion

The major finding of our study is that in addition to right atrial dilatation, the left atrial substrate is very likely to be involved in the pathogenesis of AF / AT in patients with invasively confirmed isolated precapillary PH. Our data indicates that a different arrhythmogenic mechanism is probably involved in typical right atrial arrhythmia such as type I AFL as compared to AF or other types of AT.

### Pathophysiological mechanism of AF / AT in precapillary PH

Apart from the clear mechanisms of supraventricular tachycardias (i.e. atrio-ventricular nodal reentry tachycardia or atrio-ventricular reentry tachycardia), an arrhythmogenic substrate for complex atrial arrhythmias including AF or AT in PH patients remains unclear. However, there is emerging evidence indicating a right sided substrate for complex atrial arrhythmia: PH leads to an increased afterload of the RV, resulting in RV hypertrophy and dilatation as well as upstream enlargement of the RA [14]. Long-standing PH is frequently associated with decreased conduction and tissue voltage in some cases with regions of “electrical silence” in both the RA and RV [15]. In addition, modulations of the autonomic system may trigger and perpetuate related arrhythmia [16, 17].

The mechanisms of arrhythmia have been suggested as most likely different and more similar to proarrhythmogenic substrate in left heart disease when a post-capillary component is present [8]. In cases of left heart disease, elevated end-diastolic left ventricular pressure is a well-known mechanism leading to LA structural remodeling with proarrhythmogenic effect. Left atrial remodeling, particularly LA dilatation is a well-documented risk factor for the development of AF [18, 19].

Despite the inclusion of only precapillary PH patients, increased LA diameters, LV end-diastolic diameter and higher PAWP in combination with increased RA size and elevated RAP were detected in patients with arrhythmia in our study. In addition the only change in the LA diameter in PLAX was associated with an occurrence of AF / AT in a multivariate analysis, and not with right atrial parameters. We, therefore, speculate that the left atrial substrate plays a role in arrhythmogenesis of more complex atrial tachycardias even in the presence of purely precapillary PH.

One factor possibly explaining the involvement of the left heart in the pathogenesis of AF / AT in PAH patients may be due to the definition of precapillary PH itself. PAH diagnosis is based uniquely on resting invasive pulmonary pressure measurements. In addition, the PAWP limit is set relatively high, above the limits of presumed true physiological values. This may lead to a diagnosis of purely precapillary PAH in a group of patients, in whom pulmonary hypertension is actually a combined one (combined post-capillary and precapillary PH). It has been repeatedly shown [20, 21] that a fluid challenge or exercise can unmask a postcapillary component in a large number of patients. This hypothesis would be supported by the fact, that in our study diabetes and arterial hypertension – frequent risk factors for left heart involvement with diastolic dysfunction - predicted the development of arrhythmias. As suggested by Opitz [22], these cases represent a borderline category of patients with “atypical idiopathic PAH” in whom left heart involvement remains silent under resting conditions. Finally, in borderline PAWP cases, the measurement method of PAWP may lead to an underestimation (using a digitized mean value) or overestimation (using end-expiratory values) of PAWP [23]. Our data supports the hypothesis of the participation of the truly elevated LA pressure on the development of LA substrate and its arrhythmogenity. The distribution of PAWP had negative skewness and a significant proportion of AT / AF patients contained values close to the threshold limit.

On the other hand, the high burden paroxysmal, persistent or permanent arrhythmia may be a cause of LA remodeling itself [18, 19]. Decreased atrial contraction, atrio-ventricular asynchrony, and a rapid heart rate with a reduction of diastolic filling are potential factors of left atrial remodeling. Moreover, it has been found that AF itself causes electrophysiological changes of the atrial myocardium which explains the progressive character of arrhythmia [24]. Since the LA diameter has not been significantly different according to the type of arrhythmia and is not dependent on time of onset of AT / AF, the impact of pure arrhythmia’s burden to atrial remodeling is not a simple explanation of LA enlargement in our study population. However, aging and external stressors such as arterial hypertension or diabetes, associated in our study with the presence of AF / AT were identified in our data. All those conditions are also well known factors influencing atrial electrophysiological and structural remodeling of the LA, which can be associated with the initiation of AF in the general population [25, 26] and also in the PH population [15]. These facts are well in line with our data and warrant a hypothesis of the existence of some left sided proarrhythmogenic substrate among patients with arrhythmia and precapillary PH.

Incidental findings of reduced bipolar voltages on some electro-anatomical maps of the LA support the hypothesis of the cooperation of both the left and right atrium in the pathogenesis of AF / AT in a given population. LA scarring can be detected by late enhancement magnetic resonance imaging and can be correlated well with reduced electrogram amplitudes as recorded by endocardial voltage maps [27, 28]. Atrial structural remodeling involving atrial fibrosis and scarring is a well-recognized factor in AF pathogenesis.

#### Comparison of patients with AF and AFL

Our data shows substantial differences between patients with typical right sided arrhythmia i.e. type I AFL and AF in clinical, echocardiographic and hemodynamic variables. Patients with AFL were more likely to be male, demonstrated an enlarged RA and RV, reduced RV systolic function and higher PAMP and RAP values. These cases are probably representing the consequence of true typical IPAH. Structural remodeling of the RA relating to long-standing PH with right ventricular overload and increased RV filling pressures would be prone to provoke reentry arrhythmia in the dilated RA [13, 14, 29]. Our data is in line with this observation, higher levels of RAP in patients with arrhythmia is given by higher RAP values in patient with AFL. After the exclusion of those patient with AFL, RAP is comparable between the rest of the arrhythmia group and patients without any rhythm disorder. We speculate, that the reduction of the 6-min walking test distance in the AFL subgroup is more likely a result from more advanced RA and RV dysfunction.

#### Prevalence of arrhythmia in precapillary PH

The prevalence of AF / AT in our PH population was higher than in a majority of published reports. Most retrospective and prospective studies have reported a cumulative incidence of supraventricular arrhythmia ranging from 10 to 25% in patients with PAH or inoperable CTEPH [5, 6, 8]. Only one retrospective study showed a similar (29%) cumulative incidence of arrhythmias comparable to our data in a subgroup of patients with PAH [8]. The higher prevalence of arrhythmia in the overall PH groups in our cohort is more likely given to the inclusion criteria and systematic long-lasting follow-ups for patients. According to our protocol, all patients with a detected AF / AT in their entire personal history were taken into account. Thus, our data refers to a cumulative prevalence of cases as a baseline and new case incidences together.

We detected an excessive proportion of CTEPH patients in the arrhythmia group. This prevalence is most likely given, by the high prevalence of type I AFL. An excessive proportion of AFL was detected in the CTEPH subgroup treated with PEA. Two more explanations for the increased prevalence of AFL in that group may be offered. Both advanced RA scarring resulting from RA cannulation or incision and spontaneous RA remodeling might be a plausible explanation for right sided macro-reentrant tachycardia such as AFL.

### Limitations

We must admit several limitations of our study of which the most limiting is its retrospective design. Despite a meticulous and systematic follow-up, some arrhythmias may have been missed. Our data was based on standard electrocardiograms and carefully gathered patient histories. However, due to a lack of other means of rhythm monitoring, it is likely that some self-terminating, clinically silent AF episodes might have been missed. Moreover, our hemodynamic investigation was based on a standard resting right heart catheterization which is unable to detect cases of atypical forms of PAH in whom PCWP may steeply rise during the exertion of a fluid challenge, unmasking the postcapillary component.

## Conclusion

Our study supports the role of the LA substrate in arrhythmogenesis of complex atrial arrhythmias, especially AF, despite the evidence of pure precapillary PH as diagnosed by right heart catheterization at rest. Conversely, our data suggest that the mechanism of type I AFL is more likely dependent on right atrial remodeling. An existence of both left and right atrial substrate was incidentally detected in some cases ablated with use of electro-anatomical mapping.

## Data Availability

All relevant data is in the manuscript. The datasets used are available from the corresponding author upon a reasonable request.

## Abbreviations

AF: Atrial fibrillation
AFL: Atrial flutter
AT: Atrial tachycardia
CA: Catheter ablation
CTEPH: Chronic thromboembolic pulmonary hypertension
LA: Left atrium
LV: Left ventricle
PAH: Pulmonary arterial hypertension
PAMP: Pulmonary artery mean pressure
PAWP: Pulmonary artery wedge pressure
PEA: Pulmonary endarterectomy
PH: Pulmonary hypertension
PLAX: Parasternal long axis
RA: Right atrium
RV: Right ventricle
TAPSE: Tricuspid annular plane systolic excursion
